# Evaluation of host status of garlic varieties for a plant-parasitic nematode, *Ditylenchus destructor*, by using in vitro inoculation

**DOI:** 10.5511/plantbiotechnology.24.0428a

**Published:** 2024-06-25

**Authors:** Kazuki Tadamura, Atsushi Torada, Toyoshi Yoshiga

**Affiliations:** 1 HOKUREN Agricultural Research Institute; 2 Laboratory of Nematology, Faculty of Agriculture, Saga University; 3 The United Graduate School of Agricultural Science, Kagoshima University

**Keywords:** *Allium sativum* L., host-nematode interactions, nematode inoculation

## Abstract

*Ditylenchus destructor* is a plant-parasitic nematode that severely damages garlic (*Allium sativum* L.) in Japan. *D. destructor* is detected in roots, bulbs, and outer bulb skins of garlic at harvest; however, the resistance of garlic to *D. destructor* infection is not well understood. Here, we investigated the propagation of *D. destructor* in storage organs and roots using in vitro plantlets of six Japanese garlic varieties to exclude the effects of microbes and to uniform growing conditions. In vitro inoculation can proceed simultaneously with vegetative growth, storage organ formation of garlic plantlets, and *D. destructor* infection. In ‘Fukuchi-white’, a variety susceptible to *D. destructor*, nematodes successfully propagated in storage organs and roots. Furthermore, the nematodes invaded and propagated in the newly formed storage organs. By contrast, ‘Kirishima’, ‘Hirado’, and ‘Shishimaru’ substantially suppressed more the propagation of the nematodes in storage organs and roots than ‘Fukuchi-white’. Additionally, the propagation of nematodes in newly formed storage organs was inhibited in these three varieties. ‘Shishimaru’ showed unique responses to *D. destructor* infection: nematode propagation was the lowest among six varieties in inoculation tests and the nematode-inoculated cloves turned brown. Our results suggest that several garlic varieties have resistance mechanisms that suppress the propagation of *D. destructor* in storage organs and roots, and that in vitro inoculation methods are useful for selecting resistant garlic varieties. These findings will help developing novel *D. destructor*-resistant garlic varieties and our further understanding of garlic-nematode interactions.

## Introduction

*Ditylenchus destructor* Thone, 1945 attacks many agriculturally important crops, such as potatoes (*Solanum tuberosum* L.), sweet potatoes (*Ipomoea batatas* L.), and garlic (*Allium sativum* L.), causing serious damage to crops worldwide (EPPO 2017). In Japan, *D. destructor* causes stunting in the early growing stages and severe rotting of bulbs of garlics during storage; thus, this nematode is a serious agricultural pest (Fujimura et al. 1986, 1989). *D. destructor* has been detected in the roots, bulbs, and outer bulb skins of garlic at harvest, and nematode densities in the roots and outer skins reflect the degree of bulb rot (Cheng et al. 2019). Garlic is typically grown vegetatively in the field; thus, *D. destructor* spread with infested seed cloves (Fujimura et al. 1986; Zheng et al. 2007).

*D. destructor* can also propagate by feeding on many fungal species in the soil; thus, the presence of fungi interferes with the nematodes on plants (Haraguchi and Yoshiga 2020; Kim and Ohh 1995). In addition, *D. destructor* exhibit abiotic stress tolerance to cold, desiccation, and low oxygen (Ma et al. 2020; Sugita et al. 2022). Control of *D. destructor* is difficult because of these characteristics. Several approaches for preventing damage to garlic caused by *D. destructor*, such as drying in heated storage rooms, use of thiuram-benomyl wettable powder, and soil fumigation have been attempted (Fujimura et al. 1989; Kitano and Yamashita 2011); however, nematode management remains a challenge. It is important to understand the basic mechanisms underlying nematode parasitism in garlic in order to establish a method for the nematode management.

An in vitro propagation method for *D. destructor* using field-harvested garlic cloves has been previously established (Yoshiga 2020). This method is also useful for analyzing the interactions between garlic cloves and *D. destructor*. The rotting of cloves has been developed by *D. destructor* alone and the propagation rates of the nematode differ among 16 garlic varieties cultivated in Japan (Lin et al. 2020). Furthermore, we developed an in vitro inoculation method of the nematode to garlic plantlets and improved for the garlic-nematode interaction studies (Tadamura et al. 2023). Through this method, the propagation of nematodes can be analyzed not only in storage leaves, but also in roots and foliar leaves. The physiological conditions of the host garlic plantlets were more uniform than those of the field-harvested cloves. Taken together, our nematode inoculation methods under aseptic conditions are very useful for studying plant-nematode interactions.

The aim of our study is to compare propagation of *D. destructor* among six garlic varieties in roots, storage organs, and foliar leaves, by using aseptically grown garlic plantlets, to select varieties suppressing the nematode propagation.

## Materials and methods

### Preparation of nematode inoculum

*D. destructor* strain HK1, originally isolated from garlic in Hokkaido, Japan, was used (Haraguchi and Yoshiga 2020). The method for aseptic propagation of nematodes has been described in detail previously (Yoshiga 2020). The nematodes were propagated on *Botrytis cinerea* Pers. strain SA1 growing in autoclaved barley (*Hordeum vulgare* L.) grain medium (10 g of barley grains and 10 g of distilled water) in a 100 ml glass bottle. The nematodes were collected from grain medium using a Baermann funnel (9 cm diameter, 11 cm height) (Fujiwara Scientific Co., Ltd., Tokyo, Japan) in a clean bench and left overnight. Nematodes were surface-sterilized by soaking in sterilization solution containing 0.004% mercuric chloride (Sigma-Aldrich, St. Louis, MO, USA), 0.004% sodium azide (Sigma-Aldrich), and 0.001% Triton X-100 (Nacalai Tesque, Inc., Kyoto, Japan) for 10 min then washed thrice with sterilized water by pipetting. The nematodes were then soaked in Penicillin-Streptomycin-Neomycin (PSN) antibiotic mixture (50 mg l^−1^ penicillin; 50 mg l^−1^ streptomycin; 100 mg l^−1^ neomycin) (Gibco™, Thermo Fisher Scientific Inc., Waltham, MA, USA).

### Garlic varieties

‘Fukuchi-white’ is widely grown in the cold northern regions of Japan with deep snow, including Hokkaido and Aomori (Shiga et al. 2015). The bulb of ‘Fukuchi-white’ consists of approximately six cloves, and its protective leaf is white. Previous reports have shown that *D. destructor* propagated well in ‘Fukuchi-white’ in both field and laboratory conditions, thus this variety is regarded as being susceptible to *D. destructor* (Cheng et al. 2019; Fujimura et al. 1986; Lin et al. 2020; Yoshiga 2020). The structures of field-harvested bulb and cloves were shown in Supplementary Figure S3A, B.

Hokkaido-native varieties A (Hokkaido-A) and B (Hokkaido-B) are local garlic varieties cultivated in central and southern Hokkaido, respectively, and are well-adapted to the cold northern regions of Japan. Their bulbs comprise approximately six cloves and their protective leaves are pinkish-purple. There is no information on the propagation of *D. destructor* in these varieties under field and laboratory conditions; therefore, we tested them in this study.

The ‘Kirishima’, ‘Hirado’, and ‘Shishimaru’ varieties grow mainly in the temperate southwestern part of Japan. The bulbs of ‘Kirishima’ and ‘Hirado’ consist of approximately six cloves, and their protective leaves are white with pink stripes. The bulb of ‘Shishimaru’ comprises over 15 cloves, and its protective leaf is purple. Under laboratory conditions, the propagation of *D. destructor* on field-harvested cloves in these three varieties was more suppressed than that in ‘Fukuchi-white’ (Lin et al. 2020); however, limited information is available on nematode propagation potential in roots and foliar leaves.

‘Fukuchi-white’, Hokkaido-A and Hokkaido-B plants were harvested from the same fields in our laboratory in Hokkaido. The bulbs of ‘Kirishima’ were from Kokkaen, and ‘Hirado’ and ‘Shishimaru’ from Nikko Seed Co., Ltd. All bulbs were stored at 20–35°C until use.

### Producing in vitro garlic plantlets and bulblets

Detailed methods for garlic shoot-tip culture and in vitro bulblet formation have been described previously (Tadamura et al. 2023). Briefly, field-harvested cloves were surface-sterilized with 70% ethanol (FUJIFILM Wako Pure Chemical Corporation, Osaka, Japan) for 1 min, followed by treatment with 1% sodium hypochlorite solution (FUJIFILM Wako Pure Chemical Corporation) containing 0.05% Tween 20 (FUJIFILM Wako Pure Chemical Corporation) for 10 min; the cloves were then washed three times with sterile distilled water. A shoot-tip with two leaf primordia was excised from a clove and was cultured in a plastic petri dish (9 cm diameter, 2 cm height) using Murashige and Skoog (MS) medium (Murashige and Skoog 1962) containing 3% (w/v) sucrose and 0.25% (w/v) Gelrite (FUJIFILM Wako Pure Chemical Corporation) (pH 5.8) without any plant growth regulator. The elongated shoots with rooting were transferred to a glass bottle (12 cm diameter, 15 cm height) containing fresh MS medium and were continued to culture. The shoot-tip was cultured at 25°C under 16 h light (light intensity, 45 µmol m^−2^ s^−1^)/8 h dark conditions. The in vitro garlic plantlets that were produced in a glass bottle were exposed to 5°C in the dark for 180 days. For promotion of bulblet development, the garlic plantlets in a glass bottle were transferred to a growth chamber MLR351 (PHC Corporation, Tokyo, Japan) under 16 h light (light intensity of 120 µmol m^−2^ s^−1^)/8 h dark condition at 25°C. The structures of in vitro plantlet and bulblet were shown in Supplementary Figure S3C, D, E.

### Nematode inoculation to field-harvested cloves

Surface sterilization of the cloves was performed as described above. The standard inoculation method has been described in detail previously (Lin et al. 2020; Yoshiga 2020). The basal part of the clove was cut off and a slit was made using a blade. The cloves were placed in a cylindrical glass bottle (5 cm diameter, 11 cm height) containing 20 ml of water medium solidified with 1% agar (FUJIFILM Wako Pure Chemical Corporation). An aliquot of 20 µl of the nematode suspension containing approximately 200 individuals at mixed developmental stages was inoculated onto the slit using a micropipette. The cloves were then incubated in the dark at 20°C for 56 days and cut into small pieces (<0.5 mm) using a knife and a blade. Propagated nematodes were extracted overnight using a Baermann funnel. The aqueous solution containing the isolated nematodes was appropriately diluted, and they were counted using a dissecting microscope. This procedure was repeated twice to determine the total number of nematodes. Twelve field-harvested cloves were used for the inoculation and isolation of nematodes from each garlic variety. For the inoculation test in storage leaves, sterilized cloves were cut vertically using a blade, and the internal foliar leaves were removed using tweezers. An aliquot of 20 µl of the nematode suspension containing approximately 200 individuals was dropped onto the cut surface for inoculation using a micropipette (Supplementary Figure S1A), and nematodes were incubated and isolated as described above. Nine cloves were used for the inoculation and isolation of nematodes from each garlic variety.

### Nematode inoculation test to in vitro garlic plantlets

The produced in vitro bulblets were stored in the dark at 20°C for 90 days in a plastic petri dish (9 cm diameter, 2 cm height). Thin-protective leaves were removed from the bulblets using tweezers. The bulblets were planted on 30 ml MS medium in a cylindrical glass bottle (5 cm diameter, 11 cm height) for rooting for 30 days at 20°C under 16 h light (light intensity, 45 µmol m^−2^ s^−1^)/8 h dark conditions (Supplementary Figure S3D). An aliquot of 20 µl of the nematode suspension containing approximately 200 individuals was inoculated into the contact area between the planted bulblets and MS medium using a micropipette. These plantlets were incubated at 20°C under a 16 h light (light intensity, 120 µmol m^−2^ s^−1^)/8 h dark condition. After 50 days of inoculation, 50 ml of tap water was added to the culture glass bottle and the medium was gently mixed with water using a metallic whisk to dissociate the medium from the roots. After removing the plantlets from the bottle, the medium was mixed thoroughly again, and 1 ml of the mixed medium was transferred to a Syracuse watch glass (Fujiwara Scientific Co., Ltd.), following which the number of nematodes was counted using a dissecting microscope. This procedure was repeated twice to determine the total number of nematodes in the medium. According to our previous report (Tadamura et al. 2023), nematodes isolated from the medium are regarded as the result of ectoparasitism and/or dispersion from infected roots. Garlic plantlets removed from the MS medium were separated into three parts using scissors or a blade (1) roots, 2) storage leaves, and 3) foliar leaves). The sections were then cut into small pieces using a blade. Infected nematodes were extracted by part (roots, storage leaves, and foliar leaves) overnight using a Baermann funnel, and the number of nematodes was counted using a dissecting microscope. This counting procedure was repeated twice. Twelve plantlets were used for inoculation and nematode isolation for each garlic variety.

### Nematode invasion and propagation test in newly formed in vitro garlic storage organs

In vitro bulblets were stored in the dark at 5°C for 120 days (‘Kirishima’, ‘Hirado’, and ‘Shishimaru’), 240 days (‘Fukuchi-white’), and 330 days (Hokkaido-A and Hokkaido-B) in plastic petri dishes (9 cm diameter, 2 cm height) for promoting new storage organ formation. Rooting and nematode inoculation were performed as described above. After 105 days of inoculation, the formed storage organs were harvested and rinsed with tap water to remove the nematodes from the surface. The nematodes were extracted and counted as described above. Fifteen plantlets each were used: ‘Fukuchi-white’, Kirishima’, ‘Hirado’, and ‘Shishimaru’, and nine plantlets each were used in the Hokkaido-A and Hokkaido-B. The structure of newly formed storage organ was shown in Supplementary Figure S3F.

### Statistical analysis

The mean number of nematodes isolated from storage organs, roots, foliar leaves, and medium and the standard deviation of the mean (SD) were calculated from the examined data. The Steel–Dwass test was performed to assess the differences in the mean number of isolated nematodes among the six garlic varieties.

## Results

### Propagation of *D. destructor* in field-harvested cloves

**Figure 1:**
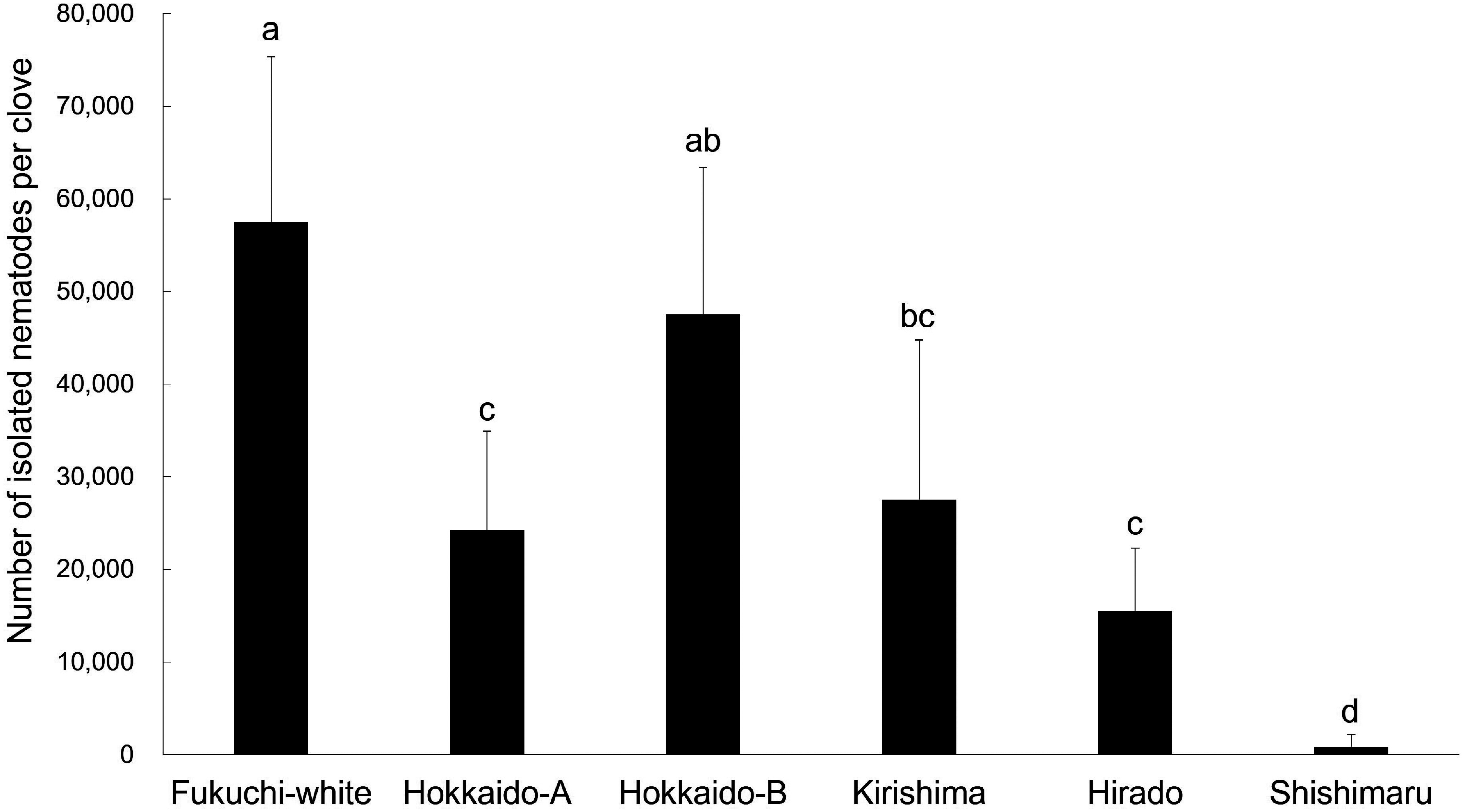
Figure 1. Number of isolated *Ditylenchus destructor* from field-harvested cloves in six garlic varieties at 56 days after inoculation. Twelve cloves per variety were used. 200 individuals of *D. destructor* were inoculated to a field-harvested clove. Bars are shown as means±standard deviation (SD) of number of isolated nematodes by the Baermann funnel method. Different letters indicate statistically significant differences (Steel–Dwass test, *p*<0.05).

**Figure 2:**
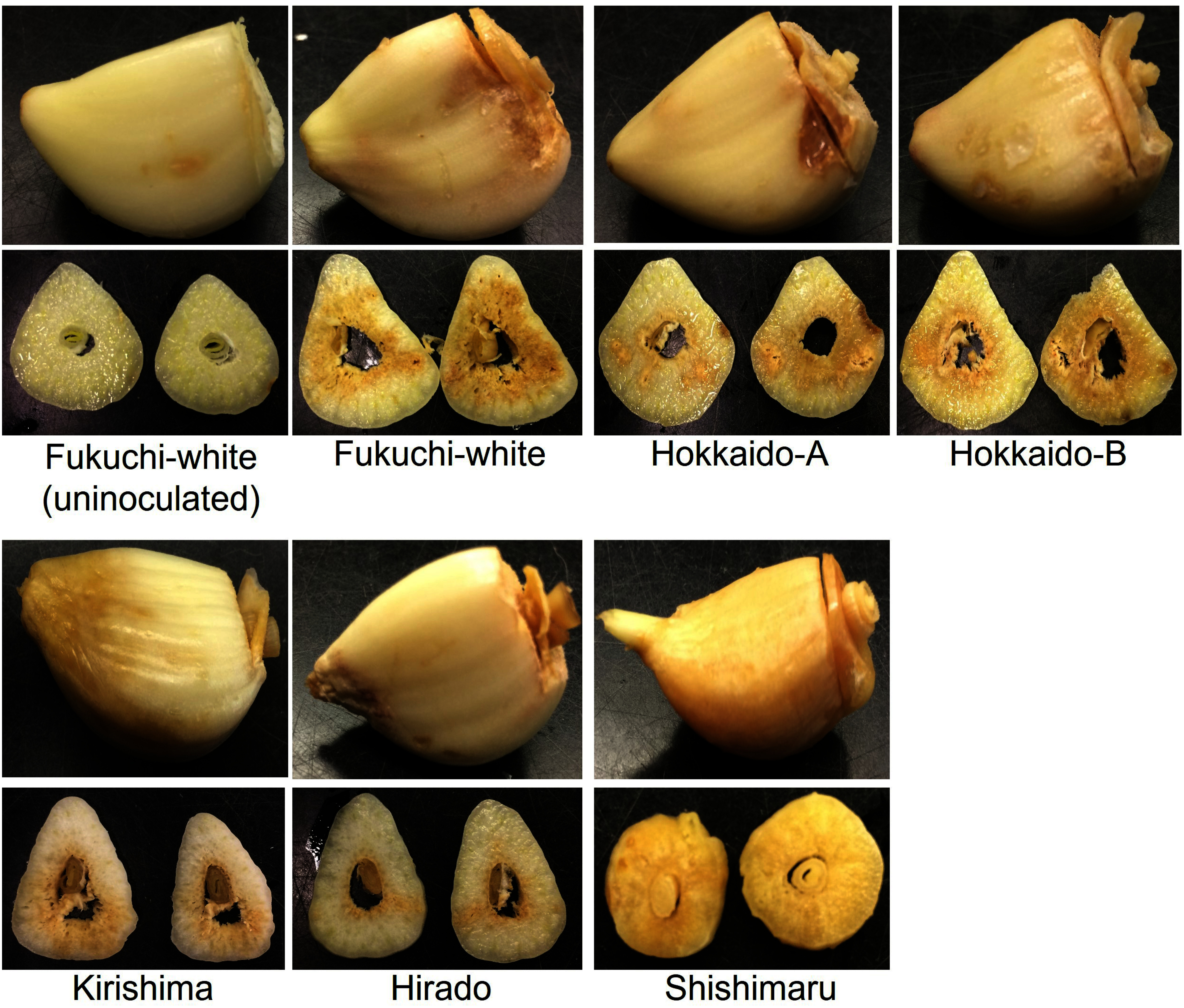
Figure 2. Comparison of *Ditylenchus destructor* inoculated cloves at 56 days after inoculation among six garlic varieties. Appearance of nematode-inoculated cloves and horizontally cut cloves is shown. Uninoculated ‘Fukuchi-white’ cloves were inoculated with sterile distilled water that did not contain any nematodes.

Nematodes were observed to have increased in all garlic varieties at 56 days after inoculation; however, the mean number of isolated nematodes differed among garlic varieties (Figure 1). The mean number of isolated nematodes in ‘Fukuchi-white’ (57,500 individuals) was the highest among varieties (Figure 1) and discolorations with void were observed in inoculated storage leaves (Figure 2). The mean number of isolated nematodes in ‘Kirishima’ (27,542 individuals), ‘Hirado’ (15,521 individuals) and ‘Shishimaru’ (847 individuals) was lower than that in ‘Fukuchi-white’ (Figure 1). Additively, areas with discoloration in ‘Kirishima’ and ‘Hirado’ were more restricted than those in ‘Fukuchi-white’ (Figure 2). The nematode-inoculated storage leaves in ‘Shishimaru’ exhibited a characteristic brown color (Figure 2). The mean number of isolated nematodes from Hokkaido-A (24,271 individuals) was lower than that from ‘Fukuchi-white’ and discoloration of storage leaves was also limited (Figures 1, 2). The mean number of isolated nematodes from Hokkaido-B was equivalent to that from Fukuchi-white (Figure 1).

We examined *D. destructor* propagation in storage leaves itself by removing foliar leaves (Supplementary Figure S1A). After 56 days of inoculation, the mean number of isolated nematodes and its differences among the six varieties were consistent with the above experiment (Figure 1, Supplementary Figure S1B). Nematode propagation was not affected by the presence or absence of foliar leaves.

Therefore, the propagation of *D. destructor* in the cloves differed among the six garlic varieties and was more suppressed in ‘Kirishima’, ‘Hirado’, ‘Shishimaru’ and Hokkaido-A than that in ‘Fukuchi-white’.

### Propagation of *D. destructor* in storage leaves, roots, and foliar leaves

**Figure 3:**
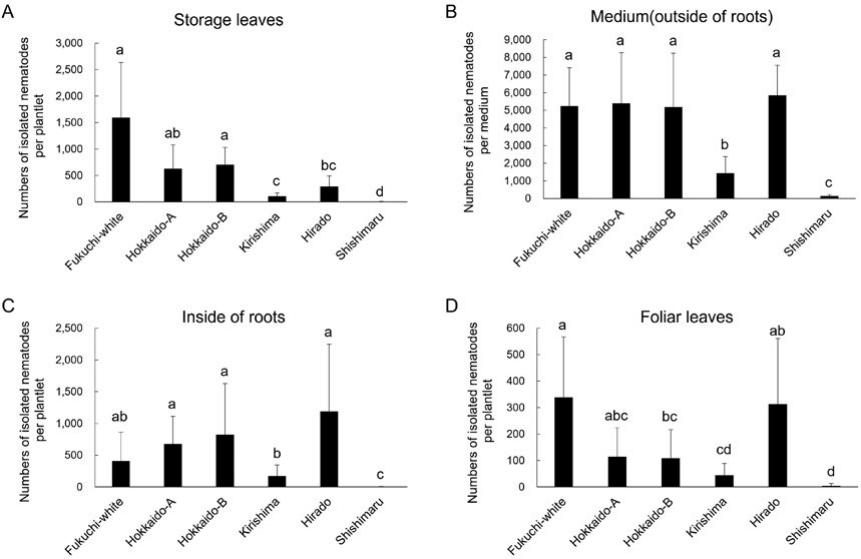
Figure 3. Number of isolated *Ditylenchus destructor* from four parts of aseptically grown plantlets in six garlic varieties at 50 days after inoculation. Twelve plantlets of each variety were used. Two hundred individuals of *D. destructor* were inoculated into the contact area between planted storage leaves (bulblets) and MS medium. Bars are shown as means±standard deviation (SD) of number of isolated nematodes by the Baermann funnel method. Different letters indicate statistically significant differences (Steel–Dwass test, *p*<0.05). Nematodes isolated from the four plant parts are shown: (A) storage leaves, (B) medium (outside the roots), (C) inside the roots, and (D) foliar leaves.

After a period of 50 days following nematode inoculation, the number of nematodes increased in all garlic varieties, but there were differences in the propagation of nematodes in the storage leaves, roots, and foliar leaves among garlic varieties (Figure 3). In storage leaves, the mean number of isolated nematodes in ‘Shishimaru’ (4 individuals), ‘Kirishima’ (108 individuals) and ‘Hirado’ (292 individuals) were lower than that in ‘Fukuchi-white’ (1,592 individuals) (Figure 3A). The nematodes isolated from storage leaves in Hokkaido-A (631 individuals) and Hokkaido-B (704 individuals) were equivalent to those in ‘Fukuchi-white’ (Figure 3A). The mean number of nematodes isolated from the medium in ‘Shishimaru’ (144 individuals) and ‘Kirishima’ (1,435 individuals) was lower than that from other garlic varieties (Figure 3B). These tendencies were similar to those observed in inside the roots (Figure 3C). The mean number of nematodes isolated from the foliar leaves was the lowest among the garlic organs in all varieties (Figure 3D). While characteristic symptoms or withering of shoots were not observed in any variety, foliar leaves and roots were elongated (Supplementary Figure S2).

### Invasion and propagation of *D. destructor* in newly formed storage organs

Due to cold treatment (5°C), all aseptically grown garlic varieties were observed to have formed new storage organs at 50 days after inoculation. In ‘Fukuchi-white’, nematodes were isolated from the medium (4,027 individuals) and inside the roots (1,007 individuals); however, nematodes were not isolated from newly formed storage organs at 50 days after inoculation (Supplementary Table S1). Thus, the nematodes did not appear to invade the newly formed storage organs at this time.

**Figure 4:**
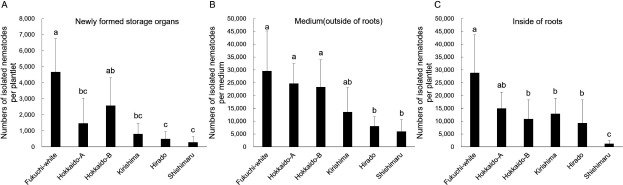
Figure 4. Number of isolated *Ditylenchus destructor* from newly formed in vitro storage organs and roots in six garlic varieties at 105 days after inoculation. Fifteen plantlets were used: ‘Fukuchi-white’, Kirishima’, ‘Hirado, and ‘Shishimaru’. Nine plantlets were used for Hokkaido-A and Hokkaido-B. Two hundred individuals of *D. destructor* were inoculated into a contact area of planted storage leaves (bulblets) and MS medium. Bars are shown as means±standard deviation (SD) of number of isolated nematodes by the Baermann funnel method. Different letters indicate statistically significant differences (Steel–Dwass test, *p*<0.05). Nematodes isolated from the three parts are shown: (A) newly formed storage organs, (B) medium (outside the roots), and (C) inside the roots.

**Figure 5:**
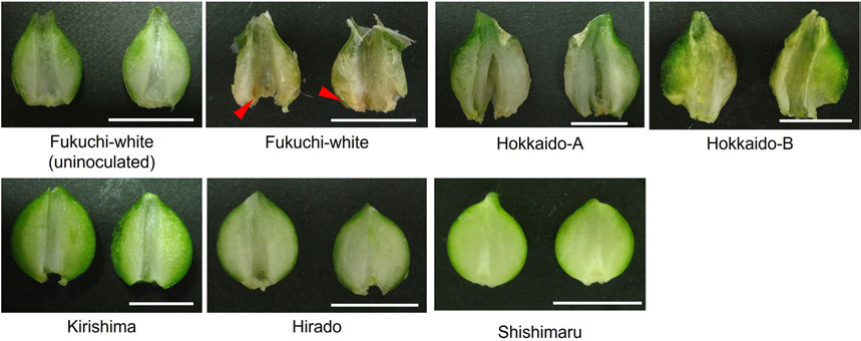
Figure 5. Comparison of newly formed in vitro bulblet among six garlic varieties after 105 days of *Ditylenchus destructor* inoculation. The newly formed in vitro bulblets were cut vertically. Scale bar indicates 1 cm. Uninoculated ‘Fukuchi-white’ cloves were dropped onto sterile distilled water without nematodes. Red arrows in ‘Fukuchi-white’ indicate brown discoloration site in storage organs.

The mean number of isolated nematodes after 105 days of inoculation from newly formed storage organs in ‘Shishimaru’ (292 individuals), ‘Hirado’ (500 individuals), ‘Kirishima’ (812 individuals), and Hokkaido-A (1,478 individuals) was lower than that in ‘Fukuchi-white’ (4,671 individuals) (Figure 4A). Newly formed storage organs in nematode-infected ‘Fukuchi-white’, Hokkaido-A and Hokkaido-B were softer than healthy individuals (Figure 5). Additively, brown discoloration was observed at the base of storage organs in ‘Fukuchi-white’ (Figure 5). However, softening and brown discoloration of newly formed storage organs were observed infrequently in ‘Kirishima’, ‘Hirado’ and ‘Shishimaru’ (Figure 5). The mean number of isolated nematodes from medium in ‘Shishimaru’ (6,042 individuals) and ‘Hirado’ (8,042 individuals) were also lower than that in ‘Fukuchi-white’ (29,583 individuals); however, their numbers in ‘Kirishima’ (13,577 individuals), Hokkaido-A (24,667 individuals), and Hokkaido-B (23,333 individuals) were equivalent to those in ‘Fukuchi-white’ (Figure 4B). The mean number of nematodes isolated from the inside of roots in ‘Shishimaru’ (1,239 individuals), ‘Hirado’ (9,333 individuals), ‘Kirishima’ (12,885 individuals), and Hokkaido-B (10,889 individuals) were lower than that in ‘Fukuchi-white’ (31,962 individuals) (Figure 4C).

Taken together, the propagation of *D. destructor* was suppressed in the newly formed storage organs in ‘Shishimaru’, ‘Kirishima’, ‘Hirado’, and Hokkaido-A. Moreover, ‘Hirado’ and ‘Shishimaru’ also suppressed the propagation of the nematodes both internally and externally from the roots.

## Discussion

*Ditylenchus destructor* is a migratory endoparasitic plant-parasitic nematode that causes serious economic damage to garlic production in Japan (Fujimura et al. 1986, 1989; Mathew and Opperman, 2020). Despite the threat posed by *D. destructor* to garlic production, the resistance of garlic to *D. destructor* infection has not been sufficiently elucidated. In the present study, we demonstrated that the propagation of *D. destructor* in aseptically grown garlic storage organs and roots differed among six Japanese garlic varieties. Moreover, the inoculation method using aseptically grown garlic plantlets has the potential to be used for selecting resistant garlic varieties.

According to previous field observations and laboratory inoculation tests using garlic cloves, ‘Fukuchi-white’ is susceptible to *D. destructor* (Cheng et al. 2019; Fujimura et al. 1986; Lin et al. 2020; Yoshiga 2020). Following the nematode inoculation method using field-harvested cloves (Lin et al. 2020; Yoshiga 2020), we also confirmed that the nematodes increased in ‘Fukuchi-white’ (Figures 1, 2). In line with these results, *D. destructor* successfully propagated in storage organs and internal and external roots in aseptically grown ‘Fukuchi-white’ plantlets (Figures 3, 4). Furthermore, we revealed that *D. destructor* could infect newly formed storage organs, causing them to rot in the absence of other organisms (Figures 4A, 5). Under inoculation conditions using aseptically grown garlic plantlets in the present study, vegetative growth, formation of new storage organs, and propagation of nematodes proceeded simultaneously in ‘Fukuchi-white’ (Figures 3, 4, 5, Supplementary Figure S2), suggesting that the inoculation method replicates the nematode infection process in the field. The growth of garlic plants in the field takes a year, and many factors, including microbes, can affect plant growth. Thus, the methods using in vitro plantlets are advantageous for anin vitroalyzing the interactions between garlic plants and *D. destructor*.

A key result of the present study was that several garlic varieties suppressed the propagation of *D. destructor* in storage organs and roots according to in vitro inoculation methods. Interestingly, ‘Shishimaru’ and ‘Kirishima’ consistently inhibited the propagation of nematodes in storage organs and roots more effectively than ‘Fukuchi-white’ (Figures 3, 4). Moreover, the propagation of nematodes in newly formed storage organs was suppressed, and rotting was not observed in either variety (Figures 4A, 5). Therefore, ‘Shishimaru’ and ‘Kirishima’ may have mechanisms to suppress the propagation of *D. destructor* at storage organs and roots. Restriction of nematode propagation in newly formed storage organs is considered to be an important trait for sustainable healthy seed production in the field to prevent the transmission of *D. destructor* by seed cloves. Furthermore, the restriction of nematode propagation in roots may contribute to the suppression of an increase in the density of the nematodes in field soil, and clove rotting during storage compared to ‘Fukuchi-white’ (Cheng et al. 2019). ‘Hirado’ also consistently suppressed the propagation of *D. destructor* in storage organs more effectively than ‘Fukuchi-white’ (Figures 3A, 4A). The nematodes were not propagated as much in the internal and external roots of ‘Hirado’ at 105 days after inoculation as in ‘Fukuchi-white’ (Figure 3B, C). However, their propagation in roots of ‘Hirado’ at 50 days after inoculation was equivalent to that in ‘Fukuchi-white’ (Figure 4B, C), which differs from the results in ‘Shishimaru’ and ‘Kirishima’.

Hokkaido-A also suppressed more the propagation of *D. destructor* in storage organs than ‘Fukuchi-white’ (Figure 4A). However, the rotting of newly formed storage organs was observed, thus its ability to suppress the nematodes may be less than that of ‘Kirishima’, ‘Hirado’, and ‘Shishimaru’ (Figure 5). Additionally, the propagation of nematodes in roots of Hokkaido-A was equivalent to that in ‘Fukuchi-white’ (Figures 3B, C, 4B, C). Totally, Hokkaido-A may have a resistance mechanism that functions only in the storage organs, which differs from that of ‘Kirishima’, ‘Hirado’, and ‘Shishimaru’. The propagation of the nematodes in storage organs and roots of Hokkaido-B was generally equivalent to that observed in ‘Fukuchi-white’ (Figures 3, 4). Even in garlic varieties for which information on nematode propagation in the field is not available, such as these local varieties, we demonstrated the potential of nematode propagation using in vitro inoculation methods.

The propagation of *D. destructor* in foliar leaves was slower than that in the other garlic organs (Figure 3). Moreover, the presence or absence of foliar leaves did not affect nematode propagation in the inoculation test using field-harvested cloves (Figure 1, Supplementary Figure S1B). Despite nematode propagation after inoculation, the foliar leaves were elongated (Supplementary Figure S2). Therefore, foliar leaves may be less suitable for nematode propagation than the storage organs or roots.

By comparing the two inoculation methods, using field-harvested garlic cloves and aseptically grown garlic plantlets, the ordinal order of the number of isolated nematodes in storage organs of each variety is consistent (Figures 1, 3A, 4A). Thus, both inoculation methods can be utilized to evaluate the propagation potential of nematodes in storage organs. However, the inoculation method using aseptically grown garlic plantlets is informative because propagation of the nematode in the roots or invasion of storage organs can also be evaluated (Figures 3, 4).

‘Shishimaru’ showed unique responses to *D. destructor* infection in the present study. The mean number of isolated nematodes in the storage organs and roots were the lowest in inoculation tests among the six garlic varieties (Figures 1, 3, 4), and the nematode-inoculated cloves showed characteristic browning (Figure 2). The protective leaves of ‘Shishimaru’ were colored purple but those of ‘Fukuchi-white’ were colored white. The pinkish-purple color of protective leaves suggests that the accumulation of anthocyanin and putative genes of phenylpropanoid pathways (i.e., *Phenylalanine ammonia lyase*, *PAL*) are increased in Mexican garlic varieties (Dufoo-Hurtado et al. 2013). *PAL* is also involved in the accumulation of phenolic compounds and in enzymatic browning (Tomas-Barberan and Espin 2001). In rice (*Oryza sativa* L.), the phenylpropanoid pathway has been suggested to be involved in the resistance mechanisms against members of the genus *Ditylenchus* (Khanam et al. 2018). In totality, garlic varieties with purple protective leaves such as ‘Shishimaru’ may activate phenylpropanoid pathways, which may be involved in the mechanisms of resistance to *D. destructor*. However, our results were limited to inoculation tests and isolation of propagated nematodes. Further experiments, such as investigation of gene expression of the phenylpropanoid pathway or gene modification analyzes, are expected to further our understanding of molecular resistance mechanisms in the future.

Garlic cross-breeding is limited because of the deficient fertility and flowering ability of garlic (Etoh 1986; Simon and Jenederek 2003; Zheng et al. 2007). Thus, tissue culture techniques are also available for generating genetic variability by somaclonal variation selection or manipulating ploidy in garlic (Dixit and Chaudhary 2014; Madhavi et al. 1991; Wen et al. 2022). Furthermore, genetic resources and landraces of garlic throughout the world may have characteristic traits, such as high yield, tolerance to biotic and abiotic stress and higher functional chemical component content; however, these genotypes are often infected with several viruses during field maintenance (Cremer et al. 2021; Hirata et al. 2016; Klukáčková et al. 2007). Shoot-tip cultures are required to eliminate viruses when using these garlic genotypes for agronomic production (Bhojwani et al. 1982). We evaluated the differences in nematode propagation in storage organs and roots using aseptically grown garlic plantlets. Therefore, in vitro inoculation methods in the present study have the potential to be used as screening techniques for garlic produced by tissue culture with unknown resistance to *D. destructor.*

In conclusion, we demonstrated that garlic varieties, such as ‘Shishimaru’, ‘Kirishima’, and ‘Hirado’ have resistance mechanisms in storage organs and roots to *D. destructor*, based on in vitro inoculation methods. These findings are expected to contribute to the development of new *D. destructor*-resistant garlic varieties for stable agricultural production and our further understanding of garlic-nematode interactions.

## Description of Supplementary Files

Supplementary Figure S1. Numbers of isolated *Ditylenchus destructor* from field harvested storage leaf itself in six garlic varieties at 56 days after inoculation.

Supplementary Figure S2. Comparison of *Ditylenchus destructor* inoculated plantlets at 50 days after inoculation among six garlic varieties.

Supplementary Figure S3. Structure of garlic plant body (variety: ‘Fukuchi-white’).

Supplementary Table S1. Number of nematodes isolated from newly formed storage organs and outside and inside of roots in ‘Fukuchi-white’ after 50 days of inoculation.

## References

[RBhojwani1982] Bhojwani SS, Cohen D, Fry PR (1982) Production of virus-free garlic and field performance of micro-propagated plants. *Sci Hortic* 18: 39–43

[RCheng2019] Cheng Z, Toyota K, Aoyama R (2019) Relationship among the potato rot nematode, *Ditylenchus destructor*, densities in soil, root and garlic (*Allium sativum*) bulbs, and rot damage in stored garlic bulbs. *Nematology* 21: 547–555

[RCremer2021] Cremer J, Campbell P, Steele V, Persley D, Thomas J, Harper S, Gambley C (2021) Detection and distribution of viruses infecting garlic crop in Australia. *Plants* 10: 1013 34069491 10.3390/plants10051013 PMC8160985

[RDixit2014] Dixit V, Chaudhary BR (2014) Colchicine-induced tetraploidy in garlic (*Allium sativum* L.) and its effect on allicin concentration. *J Hortic Sci Biotechnol* 89: 585–591

[RDufoo2013] Dufoo-Hurtado MD, Zavale-Gutiérrez KG, Cao CM, Cisneros-Zevallos L, Guevara-González RG, Torres-Pacheco I, Vázquez-Barrios ME, Rivera-Pastrana DM, Mercado-Silva EM (2013) Low-temperature conditioning of “seed” cloves enhances the expression of phenolic metabolism related genes and anthocyanin content in ‘Coreano’ garlic (*Allium sativum*) during plant development. *J Agric Food Chem* 61: 10439–10446 24164234 10.1021/jf403019t

[REPPO2017] EPPO (2017) *Ditylenchus destructor* and *Ditylenchus dipsaci*. *Bull OEPP* 47: 401–419

[REtoh1986] Etoh T (1986) Fertility of the garlic clones collected in Soviet Central Asia. *J Japan Soc Hort Sci (Engei Gakkai Zasshi)* 55: 312–319

[RFujimura1989] Fujimura T, Ichida T, Kimura T (1989) Occurrence of potato-rot nematode, *Ditylenchus destructor* Thorne, in garlic and control. 1. Evaluation of treatments applied before planting and after harvest for control. *Jpn J Nematol (Nihon Senchu Gakkai Shi)* 18: 22–29 (in Japanese with English abstract)

[RFujimura1986] Fujimura T, Washio S, Nishizawa T (1986) Garlic as a new host of the potato-rot nematode, *Ditylenchus destructor* Thorne. *Jpn J of Nematol (Nihon Senchu Gakkai Shi)* 116: 38–47 (in Japanese with English abstract)

[RHaraguchi2020] Haraguchi S, Yoshiga T (2020) Potential of the fungal feeding nematode *Aphelenchus avenae* to control fungi and the plant parasitic nematode *Ditylenchus destructor* associated with garlic. *Biol Control* 143: 104203

[RHirata2016] Hirata S, Abdelrahman M, Yamauchi N, Shigyo M (2016) Characteristics of chemical components in genetic resources of garlic *Allium sativum* collected from all over the world. *Genet Resour Crop Evol* 63: 35–45

[RKhanam2018] Khanam S, Bauters L, Singh RR, Verbeek R, Haeck A, Sultan SMD, Demeestere K, Kyndt T, Gheysen G (2018) Mechanisms of resistance in the rice cultivar Manikpukha to the rice stem nematode *Ditylenchus angustus.* *Mol Plant Pathol* 19: 1391–1402 28990717 10.1111/mpp.12622 PMC6638125

[RKim1995] Kim YH, Ohh SH (1995) *In vitro* culture and factors affecting population changes of *Ditylenchus destructor* of ginseng. *Korean J Plant Pathol* 11: 39–46

[RKitano2011] Kitano N, Yamashita K (2011) Effect of soil fumigant on damage of garlic caused by *Ditylenchus destructor.* *Ann Rept Plant Prot North Jpn (Kita Nihon Byogaityu Kenkyukai Hou)* 62: 144–147 (in Japanese)

[d67e1062] Klukáčková J, Navrátil M, Duchoslav M (2007) Natural infection of garlic (*Allium sativum* L.) by viruses in the Czech Republic. *J Plant Dis Prot* 114: 97–100

[RLin2020] Lin Y, Uchikawa H, Yoshiga T (2020) Propagation of *Ditylenchus destructor* on garlic storage leaf. *Nematol Res (Nihon Senchu Gakkaishi)* 50: 21–26

[RMa2020] Ma J, Gao B, Wang R, Li X, Chen S (2020) Transcriptome analyses of *Ditylenchus destructor* in responses to cold and desiccation stress. *Genet Mol Biol* 43: e20180057 32232317 10.1590/1678-4685-GMB-2018-0057 PMC7198036

[RMadhavi1991] Madhavi DV, Prabha TN, Singh NS, Patwardhan MV (1991) Biochemical studies with garlic (*Allium sativum* L.) cell cultures showing different flavour levels. *J Sci Food Agric* 56: 15–24

[RMathew2020] Mathew R, Opperman CH (2020) Current insights into migratory endoparasitism: Deciphering the biology, parasitism mechanisms, and management strategies of key migratory endo parasitic phytonematodes. *Plants* 9: 671 32466416 10.3390/plants9060671 PMC7356796

[RMurashige1962] Murashige T, Skoog FA (1962) A revised medium for rapid growth and bio assays with tobacco tissue culture. *Physiol Plant* 15: 473–497

[RShiga2015] Shiga Y, Tsutsui S, Mikami T (2015) Morphological characteristics and ancestry of Japanese garlic clones: An overview. *J Appl Hortic* 17: 210–212

[RSimon2003] Simon PW, Jenederek MM (2003) Flowering, seed production, and the genesis of garlic breeding. In: Janick J (ed) *Plant Breeding Reviews*. Wiley, New York, pp 211–244

[RSugita2022] Sugita Y, Sobagaki T, Yoshiga T (2022) Low-oxygen tolerance of *Ditylenchus destructor* (Tylenchida: Anguinidae). *Appl Entomol Zool* 57: 131–136

[RTadamura2023] Tadamura K, Mori D, Torada A, Yoshiga T (2023) Ecto- and endo-parasitism of *Ditylenchus destructor* on garlic roots. *Nematology* 25: 1073–1077

[RTomas2001] Tomás-Barberán FA, Espin JC (2001) Phenolic compounds and related enzymes as determinants of quality in fruits and vegetables. *J Sci Food Agric* 81: 853–876

[RWen2022] Wen Y, Liu H, Meng H, Qiao L, Zhang G, Cheng Z (2022) *In vitro* induction and phenotypic variations of autotetraploid garlic (*Allium sativum* L.) with dwarfism. *Front Plant Sci* 13: 917910 35812906 10.3389/fpls.2022.917910 PMC9258943

[RYoshiga2020] Yoshiga T (2020) *In vitro* culture method of *Ditylenchus destructor* (Tylenchida: Anguinidae) using garlic. *Appl Entomol Zool* 55: 435–437

[RZheng2007] Zheng SJ, Kamenetsky R, Féréol L, Barnandiaran X, Rabinowithch HD, Chovelon V, Kik C (2007) Garlic breeding system innovations. *Med Aromat Plant Sci Biotechnol* 1: 6–15

