# Supplementary material for: Evaluation of host status of garlic varieties for a plant-parasitic nematode, Ditylenchus destructor, by using in vitro inoculation
Source: Plant Biotechnol (Tokyo). 2024 Jun 25;41(2):137–45. doi: 10.5511/plantbiotechnology.24.0428a (PMC11500592; doi:10.5511/plantbiotechnology.24.0428a)
Supplement: Supplementary Data [file plantbiotechnology-41-2-24.0428a-s001.pdf]

Evaluation of host status of garlic varieties for a plant-parasitic nematode, *Ditylenchus destructor*, by using *in vitro* inoculation

Kazuki Tadamura[1,2,3*], Atsushi Torada[1], Toyoshi Yoshiga[2,3]

[1]HOKUREN Agricultural Research Institute, Naganuma, Hokkaido 069-1317, Japan
[2]Laboratory of Nematology, Faculty of Agriculture, Saga University, Saga 840-8502, Japan
[3]The United Graduate School of Agricultural Science, Kagoshima University, Kagoshima 890-0065, Japan

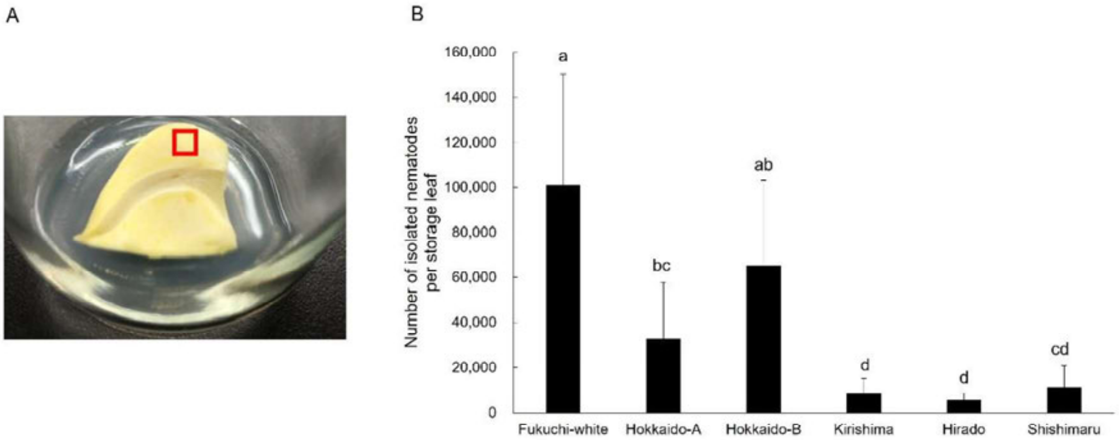

Supplementary Figure S1. Numbers of isolated *Ditylenchus destructor* from field harvested storage leaf itself in six garlic varieties at 56 days after inoculation.
(A) Prepared storage leaves for inoculation were vertically cut, and the foliar leaves were removed. Red box indicates the nematode inoculation area. (B) Nine storage leaves from each variety were used. Two hundred individuals of *D. destructor* were inoculated into the prepared storage leaves. Bars are shown as means ± standard deviation (SD) of number of isolated nematodes by the Baermann funnel method. Different letters indicate statistically significant differences (Steel–Dwass test, $P<0.05$).

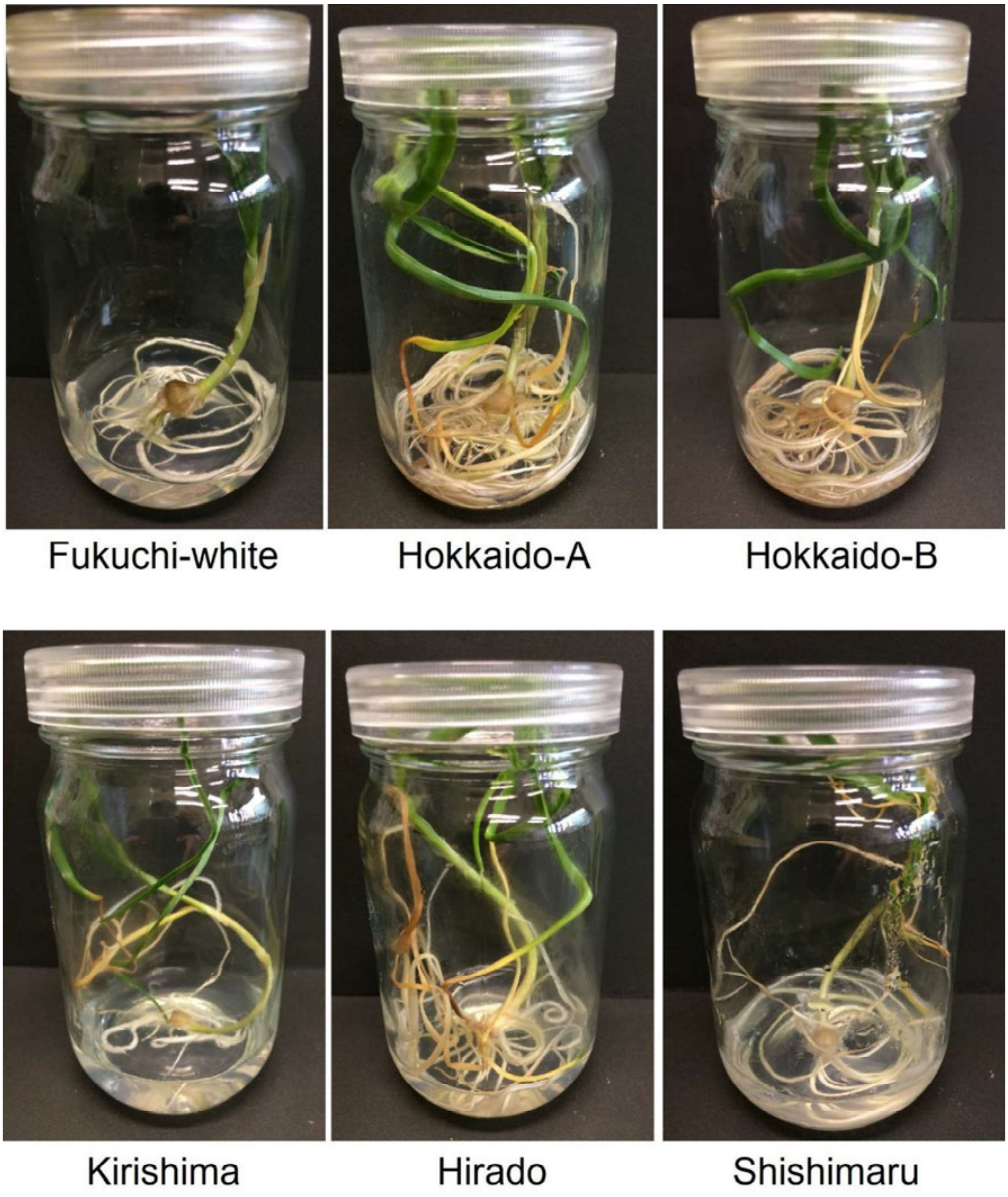

Supplementary Figure S2. Comparison of *Ditylenchus destructor* inoculated plantlets at 50 days after inoculation among six garlic varieties.

The nematode-inoculated plantlets. There are no distinctive symptoms in any of the varieties.

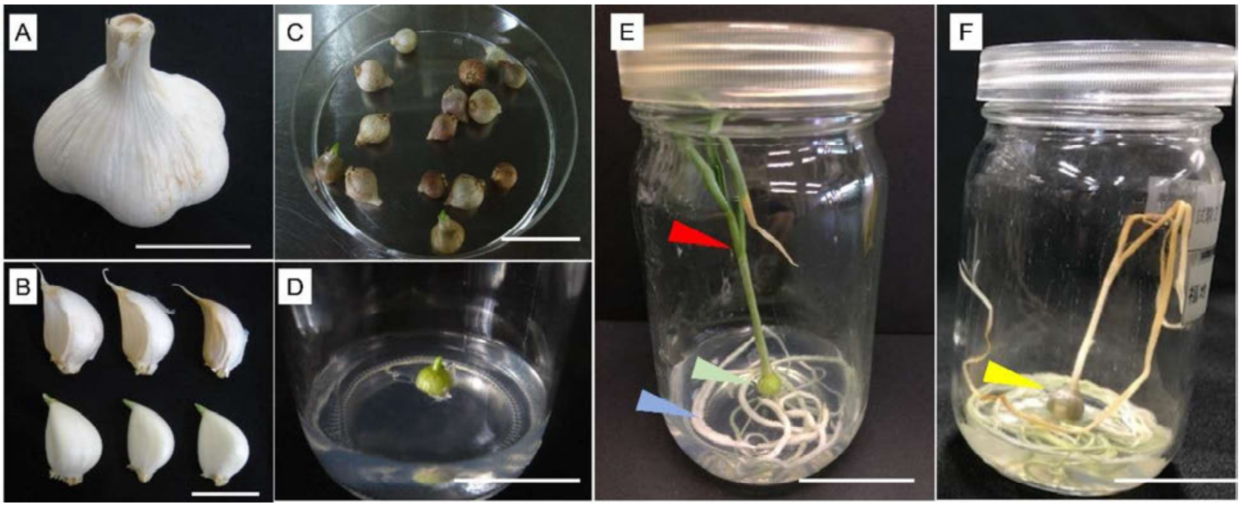

Supplementary Figure S3. Structure of garlic plant body (variety: 'Fukuchi-white').

(A) Field-harvested bulb. The bulb consists approximately six cloves, which are covered with outer skin. (B) Field-harvested cloves. The cloves in the upper row are covered with protective leaf and outer skin. The cloves removing the protective leaf and outer skin are shown in the bottom row. (C) Bulblets produced *in vitro* condition. The bulblet is consisted of storage leaf and primordia of roots and leaf. The bulblet can germinate and be rooting as well as field-harvested cloves. (D) After planting the bulblet in the MS medium. (E) *In vitro* plantlet. The whole plant body cultured in glass bottle is described as plantlet in the present study. The *in vitro* plantlet is consisted foliar leaf (red arrow), storage leaf (green arrow), and roots (blue arrow). (F) Newly formed storage organ. Cold treatment of bulblet prior to planting encourages the formation of new storage organ under *in vitro* condition. The tissue is shown newly formed storage organs (yellow arrow) in the present study. All scale bars in the figure indicate 3 cm.

Supplementary Table S1. Number of nematodes isolated from newly formed storage organs and outside and inside of roots in 'Fukuchi-white' after 50 days of inoculation

Individuals of garlic plantlets	Numbers of nematodes isolated from		
	Newly formed storage organs	Medium (outside of roots)	Inside of roots
1	0	6,260	1,480
2	0	3,840	800
3	0	1,980	740
Mean	0	4,027	1,007
Standard deviation of the mean	0	2,146	411
